# Host factors are associated with vaginal microbiome structure in pregnancy in the ECHO Cohort Consortium

**DOI:** 10.1038/s41598-024-62537-7

**Published:** 2024-05-23

**Authors:** Kimberly McKee, Christine M. Bassis, Jonathan Golob, Beatrice Palazzolo, Ananda Sen, Sarah S. Comstock, Christian Rosas-Salazar, Joseph B. Stanford, Thomas O’Connor, James E. Gern, Nigel Paneth, Anne L. Dunlop, ECHO Cohort Consortium, P. Brian Smith, L. Kristin Newby, Linda Adair, Lisa P. Jacobson, Diane Catellier, Monica McGrath, Christian Douglas, Priya Duggal, Emily Knapp, Amii Kress, Courtney K. Blackwell, Maxwell A. Mansolf, Jin-Shei Lai, Emily Ho, David Cella, Richard Gershon, Michelle L. Macy, Suman R. Das, Jane E. Freedman, Simon A. Mallal, John A. McLean, Ravi V. Shah, Meghan H. Shilts, Akram N. Alshawabkeh, Jose F. Cordero, John Meeker, Leonardo Trasande, Carlos A. Camargo, Kohei Hasegawa, Zhaozhong Zhu, Ashley F. Sullivan, Dana Dabelea, Wei Perng, Traci A. Bekelman, Greta Wilkening, Sheryl Magzamen, Brianna F. Moore, Anne P. Starling, Deborah J. Rinehart, Daphne Koinis Mitchell, Viren D’Sa, Sean C. L. Deoni, Hans-Georg Mueller, Cristiane S. Duarte, Catherine Monk, Glorisa Canino, Jonathan Posner, Tenneill Murray, Claudia Lugo-Candelas, Anne L. Dunlop, Patricia A. Brennan, Christine Hockett, Amy Elliott, Assiamira Ferrara, Lisa A. Croen, Monique M. Hedderson, John Ainsworth, Leonard B. Bacharier, Casper G. Bendixsen, James E. Gern, Diane R. Gold, Tina V. Hartert, Daniel J. Jackson, Christine C. Johnson, Christine L. M. Joseph, Meyer Kattan, Gurjit K. Khurana Hershey, Robert F. Lemanske, Susan V. Lynch, Rachel L. Miller, George T. O’Connor, Carole Ober, Dennis Ownby, Katherine Rivera-Spoljaric, Patrick H. Ryan, Christine M. Seroogy, Anne Marie Singh, Robert A. Wood, Edward M. Zoratti, Rima Habre, Shohreh Farzan, Frank D. Gilliland, Irva Hertz-Picciotto, Deborah H. Bennett, Julie B. Schweitzer, Rebecca J. Schmidt, Janine M. LaSalle, Alison E. Hipwell, Kate E. Keenan, Catherine J. Karr, Nicole R. Bush, Kaja Z. LeWinn, Sheela Sathyanarayana, Qi Zhao, Frances Tylavsky, Kecia N. Carroll, Christine T. Loftus, Leslie D. Leve, Jody M. Ganiban, Jenae M. Neiderhiser, Scott T. Weiss, Augusto A. Litonjua, Cindy T. McEvoy, Eliot R. Spindel, Robert S. Tepper, Craig J. Newschaffer, Kristen Lyall, Heather E. Volk, Rebecca Landa, Sally Ozonoff, Joseph Piven, Heather Hazlett, Juhi Pandey, Robert Schultz, Steven Dager, Kelly Botteron, Daniel Messinger, Wendy Stone, Jennifer Ames, Thomas G. O’Connor, Richard K. Miller, Emily Oken, Michele R. Hacker, Tamarra James-Todd, T. Michael O’Shea, Rebecca C. Fry, Jean A. Frazier, Rachana Singh, Caitlin Rollins, Angela Montgomery, Ruben Vaidya, Robert M. Joseph, Lisa K. Washburn, Semsa Gogcu, Kelly Bear, Julie V. Rollins, Stephen R. Hooper, Genevieve Taylor, Wesley Jackson, Amanda Thompson, Julie Daniels, Michelle Hernandez, Kun Lu, Michael Msall, Madeleine Lenski, Rawad Obeid, Steven L. Pastyrnak, Elizabeth Jensen, Christina Sakai, Hudson Santos, Jean M. Kerver, Nigel Paneth, Charles J. Barone, Michael R. Elliott, Douglas M. Ruden, Chris Fussman, Julie B. Herbstman, Amy Margolis, Susan L. Schantz, Sarah Dee Geiger, Andrea Aguiar, Karen Tabb, Rita Strakovsky, Tracey Woodruff, Rachel Morello-Frosch, Amy Padula, Joseph B. Stanford, Christina A. Porucznik, Angelo P. Giardino, Rosalind J. Wright, Robert O. Wright, Brent Collett, Nicole Baumann-Blackmore, Ronald Gangnon, Daniel J. Jackson, Chris G. McKennan, Jo Wilson, Matt Altman, Judy L. Aschner, Annemarie Stroustrup, Stephanie L. Merhar, Paul E. Moore, Gloria S. Pryhuber, Mark Hudak, Ann Marie Reynolds Lyndaker, Andrea L. Lampland, Burton Rochelson, Sophia Jan, Matthew J. Blitz, Michelle W. Katzow, Zenobia Brown, Codruta Chiuzan, Timothy Rafael, Dawnette Lewis, Natalie Meirowitz, Brenda Poindexter, Tebeb Gebretsadik, Sarah Osmundson, Jennifer K. Straughen, Amy Eapen, Andrea Cassidy-Bushrow, Ganesa Wegienka, Alex Sitarik, Kim Woodcroft, Audrey Urquhart, Albert Levin, Tisa Johnson-Hooper, Brent Davidson, Tengfei Ma, Emily S. Barrett, Martin J. Blaser, Maria Gloria Dominguez-Bello, Daniel B. Horton, Manuel Jimenez, Todd Rosen, Kristy Palomares, Lyndsay A. Avalos, Yeyi Zhu, Kelly J. Hunt, Roger B. Newman, Michael S. Bloom, Mallory H. Alkis, James R. Roberts, Sunni L. Mumford, Heather H. Burris, Sara B. DeMauro, Lynn M. Yee, Aaron Hamvas, Antonia F. Olidipo, Andrew S. Haddad, Lisa R. Eiland, Nicole T. Spillane, Kirin N. Suri, Stephanie A. Fisher, Jeffrey A. Goldstein, Leena B. Mithal, Raye-Ann O. DeRegnier, Nathalie L. Maitre, Ruby H. N. Nguyen, Meghan M. JaKa, Abbey C. Sidebottom, Michael J. Paidas, JoNell E. Potter, Natale Ruby, Lunthita Duthely, Arumugam Jayakumar, Karen Young, Isabel Maldonado, Meghan Miller, Jonathan L. Slaughter, Sarah A. Keim, Courtney D. Lynch, Kartik K. Venkatesh, Kristina W. Whitworth, Elaine Symanski, Thomas F. Northrup, Hector Mendez-Figueroa, Ricardo A. Mosquera, Margaret R. Karagas, Juliette C. Madan, Debra M. MacKenzie, Johnnye L. Lewis, Brandon J. Rennie, Bennett L. Leventhal, Young Shin Kim, Somer Bishop, Sara S. Nozadi, Li Luo, Barry M. Lester, Carmen J. Marsit, Todd Everson, Cynthia M. Loncar, Elisabeth C. McGowan, Stephen J. Sheinkopf, Brian S. Carter, Jennifer Check, Jennifer B. Helderman, Charles R. Neal, Lynne M. Smith

**Affiliations:** 1 Department of Family Medicine, University of Michigan, 1018 Fuller St, Ann Arbor, MI 48104 USA; 2 Division of Infectious Diseases, Department of Internal Medicine, University of Michigan, Ann Arbor, MI USA; 3 Department of Food Science and Human Nutrition, Michigan State University, East Lansing, MI USA; 4 Department of Pediatrics, Vanderbilt University Medical Center, Nashville, TN USA; 5 Department of Family and Preventive Medicine, University of Utah School of Medicine, Salt Lake City, UT USA; 6 Departments of Neuroscience and Obstetrics & Gynecology, University of Rochester, Rochester, NY USA; 7 Department of Pediatrics, University of Wisconsin, Madison, WI USA; 8 Departments of Epidemiology & Biostatistics and Pediatrics & Human Development, Michigan State University, East Lansing, MI USA; 9 Department of Gynecology and Obstetrics, Emory University School of Medicine, Atlanta, GA USA; 10 Division of Neonatology, Department of Pediatrics, Duke Clinical Research Institute, Duke University School of Medicine, Durham, NC USA; 11 Division of Cardiology, Department of Medicine, Duke Clinical Research Institute, Duke University School of Medicine, Durham, NC USA; 12 Department of Nutrition, Gillings School of Global Public Health, University of North Carolina at Chapel Hill, Chapel Hill, NC USA; 13 Department of Epidemiology, Johns Hopkins University, Bloomberg School of Public Health, Baltimore, MD USA; 14 Research Triangle Institute, Research Triangle Park, NC USA; 15 Department of Medical Social Sciences, Feinberg School of Medicine, Northwestern University, Chicago, IL USA; 16 Department of Pediatrics, Feinberg School of Medicine, Northwestern University and Ann & Robert H. Lurie Children’s Hospital of Chicago, Chicago, IL USA; 17 Division of Infectious Diseases, Department of Medicine, Vanderbilt University Medical Center, Nashville, TN USA; 18 Division of Cardiovascular Medicine, Department of Medicine, Vanderbilt University Medical Center, Nashville, TN USA; 19 Department of Chemistry, Vanderbilt University, Nashville, TN USA; 20 College of Engineering, Northeastern University, Boston, MA USA; 21 College of Public Health, Department of Epidemiology & Biostatistics, University of Georgia, Athens, GA USA; 22 Environmental Health Sciences, School of Public Health, University of Michigan, Ann Arbor, MI USA; 23 Departments of Pediatrics and Population Health, NYU Grossman School of Medicine, New York, NY USA; 24 Department of Emergency Medicine, Massachusetts General Hospital, Harvard Medical School, Boston, MA USA; 25 Lifecourse Epidemiology of Adiposity and Diabetes (LEAD) Center, University of Colorado Anschutz Medical Campus, Aurora, CO USA; 26 Environmental and Radiological Health Sciences, Colorado School of Public Health, Colorado State University, Fort Collins, CO USA; 27 Epidemiology, University of North Carolina at Chapel Hill, Chapel Hill, NC USA; 28 Center for Health Systems Research, Denver Health and Hospital Authority, Denver, CO USA; 29 Department of Pediatrics, Rhode Island Hospital, The Alpert Medical School of Brown University, Providence, Rhode Island, USA; 30 Division of Gender Equality, Maternal, Newborn & Child Health Discovery & Tools Team, Bill & Melinda Gates Foundation, Seattle, WA USA; 31 Department of Statistics, University of California, Davis, Davis, CA USA; 32 Division of Child and Adolescent Psychiatry, Columbia University-NYSPI, New York, NY USA; 33 Department of Obstetrics & Gynecology, Columbia University-NYSPI, New York, NY USA; 34 Behavioral Sciences Research Institute, University of Puerto Rico, School of Medicine, Rio Piedras, Puerto Rico; 35 Child & Family Mental Health & Community Psychiatry Division, Duke University School of Medicine, Duke Psychiatry & Behavioral Sciences, Durham, NC USA; 36 Department of Psychology, Emory University, Atlanta, GA USA; 37 Department of Pediatrics, Avera Research Institute, University of South Dakota School of Medicine, Rapid City, Sioux Falls, SD USA; 38 Department of Pediatrics , Avera Research Institute, University of South Dakota School of Medicine, Sioux Falls, SD USA; 39 Division of Research, Kaiser Permanente Northern California, Oakland, CA USA; 40 Centre for Health Informatics, University of Manchester, Manchester, UK; 41 Department of Pediatrics, Monroe Carell Jr Children’s Hospital at Vanderbilt, Vanderbilt University Medical Center, Nashville, TN USA; 42 National Farm Medicine Center, Marshfield Clinic Research Institute, Marshfield, WI USA; 43 Department of Pediatrics, University of Wisconsin School of Medicine and Public Health, Madison, WI USA; 44 The Channing Division of Network Medicine; Department of Medicine, Brigham and Women’s Hospital, Harvard Medical School, Boston, MA USA; 45 Division of Pediatric Allergy, Immunology, and Pulmonary Medicine, Department of Medicine, Department of Pediatrics, Vanderbilt University Medical Center, Nashville, TN USA; 46 Department of Public Health Sciences, Henry Ford Health, Detroit, MI USA; 47 Department of Pediatrics, Columbia University Medical Center, New York, NY USA; 48 Division of Asthma Research, Cincinnati Children’s Hospital Medical Center, Cincinnati, OH USA; 49 Department of Medicine, University of California, San Francisco, CA USA; 50 Department of Medicine; Division of Clinical Immunology, Icahn School of Medicine at Mount Sinai, New York, NY USA; 51 Department of Pediatrics, Boston University School of Medicine, Boston, MA USA; 52 Department of Human Genetics, University of Chicago, Chicago, IL USA; 53 Department of Pediatrics, Washington University School of Medicine, St Louis, MO USA; 54 Department of Pediatrics and College of Medicine, Division of Biostatistics and Epidemiology, University of Cincinnati, Cincinnati, OH USA; 55 Department of Pediatrics, Johns Hopkins University School of Medicine, Baltimore, MD USA; 56 Division of Allergy and Clinical Immunology, Henry Ford Health, Detroit, MI USA; 57 Department of Population and Public Health Sciences, University of Southern California, Los Angeles, CA USA; 58 MIND Institute and Department of Public Health Sciences, University of California, Davis, Davis, CA USA; 59 Department of Public Health Sciences, University of California, Davis, Davis, CA USA; 60 Department of Psychiatry and Behavioral Science and the MIND Institute, University of California, Davis, Davis, CA USA; 61 Medical Microbiology and Immunology; MIND Institute, University of California, Davis, Davis, CA USA; 62 Psychiatry and Psychology, University of Pittsburgh, Pittsburgh, PA USA; 63 Psychiatry and Behavioral Neuroscience, University of Chicago, Chicago, IL USA; 64 Department of Pediatrics, School of Medicine, Department of Environmental and Occupational Health Sciences, School of Public Health, University of Washington, Seattle, WA USA; 65 Department of Psychiatry and Behavioral Sciences and Department of Pediatrics, School of Medicine, University of California, San Francisco, San Francisco, CA USA; 66 Department of Psychiatry and Behavioral Sciences, School of Medicine, University of California, San Francisco, San Francisco, CA USA; 67 Department of Pediatrics, School of Medicine; Department of Environmental and Occupational Health Sciences, School of Public Health, University of Washington and Seattle Children’s Research Institute, Seattle, WA USA; 68 Department of Preventive Medicine, University of Tennessee Health Science Center, Memphis, TN USA; 69 Department of Pediatrics, Department of Environmental Medicine & Public Health, Icahn School of Medicine at Mount Sinai, New York, NY USA; 70 Department of Environmental and Occupational Health Sciences; School of Public Health, University of Washington, Seattle, WA USA; 71 Department of Counseling Psychology and Human Services & Prevention Science Institute, University of Oregon, Eugene, OR USA; 72 Department of Psychological and Behavioral Sciences, George Washington University, Washington, DC USA; 73 Department of Psychology, Penn State University, University Park, PA USA; 74 Channing Division of Network Medicine, Department of Medicine, Brigham and Women’s Hospital and Harvard Medical School, Boston, MA USA; 75 Pediatric Pulmonary Division, Department of Pediatrics, Golisano Children’s Hospital, University of Rochester, Rochester, NY USA; 76 Division of Neonatology, Department of Pediatrics, Oregon Health & Science University, Portland, OR USA; 77 Division of Neuroscience, Oregon National Primate Research Center, Beaverton, OR USA; 78 Division of Pediatric Pulmonology, Department of Pediatrics, Indiana School of Medicine, Indianapolis, IN USA; 79 College of Health and Human Development, Penn State, State College, PA USA; 80 AJ Drexel Autism Institute, Drexel University, Philadelphia, PA USA; 81 Mental Health, Johns Hopkins University, Baltimore, MD USA; 82 Department of Psychiatry and Behavioral Sciences, Center for Autism and Related Disorders, Kennedy Krieger Institute, Johns Hopkins University, Baltimore, MD USA; 83 MIND Institute, Department of Psychiatry, University of California Davis, Sacramento, CA USA; 84 Department of Psychiatry, University of North Carolina, Chapel Hill, NC USA; 85 Center for Autism Research, Children’s Hospital of Philadelphia, Philadelphia, PA USA; 86 Department of Radiology, University of Washington, Seattle, WA USA; 87 Department of Psychiatry, Washington University, St Louis, MO USA; 88 Department of Psychology, University of Miami, Miami, FL USA; 89 Department of Psychology, University of Washington, Seattle, WA USA; 90 Kaiser Permanente Division of Research, Kaiser Permanente, Oakland, CA USA; 91 Departments of Psychiatry, Neuroscience, Obstetrics and Gynecology, University of Rochester, Rochester, NY USA; 92 Departments of Obstetrics and Gynecology, University of Rochester, Rochester, NY USA; 93 Division of Chronic Disease Research Across the Lifecourse, Department of Population Medicine, Harvard Pilgrim Health Care Institute and Harvard Medical School, Boston, MA USA; 94 Department of Obstetrics and Gynecology, Beth Israel Deaconess Medical Center, Boston, MA USA; 95 Department of Environmental Health, Harvard Chan School of Public Health, Boston, MA USA; 96 Division of Neonatology, Department of Pediatrics, University of North Carolina School of Medicine, Chapel Hill, NC USA; 97 Department of Environmental Sciences and Engineering, University of North Carolina Gillings School of Global Public Health, Chapel Hill, NC USA; 98 EK Shriver Center and Psychiatry, UMASS Chan Medical School, Worcster, MA USA; 99 Department of Pediatrics, Tufts University School of Medicine, Boston, MA USA; 100 Department of Neurology, Harvard Medical School, Boston, MA USA; 101 Division of Neonatology, Department of Pediatrics, Yale School of Medicine, New Haven, CT USA; 102 Department of Pediatrics, University of Massachusetts Chan Medical School-Baystate, Springfield, MA USA; 103 Department of Anatomy & Neurobiology, Boston University Chobanian & Avedisian School of Medicine, Boston, MA USA; 104 Pediatrics, Wake Forest School of Medicine, Winston-Salem, NC USA; 105 Section of Neonatology, Department of Pediatrics; Department of Pediatrics, Wake Forest School of Medicine, Wake Forest University School of Medicine/Atrium Health Wake Forest, Winston-Salem, NC USA; 106 Section of Neonatology, Department of Pediatrics, ECU Health, Greenville, NC USA; 107 Department of Health Sciences, School of Medicine, University of North Carolina at Chapel Hill, Chapel Hill, NC USA; 108 Pediatrics, School of Medicine, University of North Carolina at Chapel Hill, Chapel Hill, NC USA; 109 Department of Anthropology, Department of Nutrition, University of North Carolina at Chapel Hill, Gillings School of Global Public Health, University of North Carolina at Chapel Hill, Chapel Hill, NC USA; 110 Epidemiology and Maternal and Child Health, University of North Carolina at Chapel Hill, Gillings School of Global Public Health, University of North Carolina at Chapel Hill, Chapel Hill, NC USA; 111 Environmental Sciences and Engineering, Gillings School of Global Public Health, University of North Carolina at Chapel Hill, Chapel Hill, NC USA; 112 Kennedy Research Center On Intellectual and Neurodevelopmental Disabilities, University of Chicago Medicine: Comer Children’s Hospital, Chicago, IL USA; 113 Department of Epidemiology and Biostatistics, Michigan State University, East Lansing, MI USA; 114 Pediatrics, Beaumont Hospital, Royal Oak, MI USA; 115 Pediatrics, Corewell Health, Helen DeVos Children’s Hospital, Grand Rapids, MI USA; 116 Epidemiology and Prevention, Wake Forest University School of Medicine, Winston-Salem, NC USA; 117 Pediatrics, Mass General Hospital for Children, Boston, MA USA; 118 Dean’s Office Graduate School, School of Nursing and Health Studies, University of Miami, Coral Gables, FL USA; 119 Departments of Epidemiology & Biostatistics, and Pediatrics & Human Development, Michigan State University, College of Human Medicine, East Lansing, MI USA; 120 Department of Pediatrics, Henry Ford Health, Detroit, MI USA; 121 Department of Biostatistics, University of Michigan, Ann Arbor, MI USA; 122 Department of Obstetrics and Gynecology, Institute of Environmental Health Sciences (IEHS), C.S. Mott Center for Human Health and Development, Wayne State University, Detroit, MI USA; 123 Lifecourse Epidemiology and Genomics Division, Michigan Department of Health and Human Services (MDHHS), Lansing, MI USA; 124 Department of Environmental Health Sciences, Columbia University Mailman School of Public Health, New York, NY USA; 125 Department of Psychiatry, Columbia University Irving Medical Center, New York, NY USA; 126 Beckman Institute for Advanced Science and Technology, Department of Comparative Biosciences, University of Illinois Urbana-Champaign, Urbana, IL USA; 127 Beckman Institute for Advanced Science and Technology, Department of Kinesiology and Community Health, University of Illinois Urbana-Champaign, Urbana, IL USA; 128 Beckman Institute for Advanced Science and Technology, Department of Social Work, University of Illinois Urbana-Champaign, Urbana, IL USA; 129 Program on Reproductive Health and the Environment, University of California, San Francisco, San Francisco, CA USA; 130 Department of Environmental Science, Policy and Management and School of Public Health, University of California, Berkeley, Berkeley, CA USA; 131 Department of Family and Preventive Medicine, Spencer Fox Eccles School of Medicine, University of Utah, Salt Lake City, UT USA; 132 Department of Pediatrics, Spencer Fox Eccles School of Medicine, University of Utah, Salt Lake City, UT USA; 133 Department of Environmental Medicine & Public Health, Icahn School of Medicine at Mount Sinai, New York, NY USA; 134 Department of Psychiatry and Behavioral Medicine, University of Washington, Seattle Children’s Research Institute, Seattle, WA USA; 135 Department of Population Health Sciences, University of Wisconsin, Madison, WI USA; 136 Department of Statistics, University of Pittsburgh, Pittsburgh, PA USA; 137 Department of Medicine, University of Washington, Seattle, WA USA; 138 Department of Pediatrics, Albert Einstein College of Medicine, Hackensack Meridian School of Medicine, Center for Discovery and Innovation , Bronx, Nutley, NJ USA; 139 Department of Pediatrics, Northwell Health, Cohen Children’s Medical Center, and the Zucker School of Medicine at Hofstra/Northwell, New Hyde Park, NY USA; 140 Department of Pediatrics, Cincinnati Children’s, Cincinnati, OH USA; 141 Department of Pediatrics, University of Rochester Medical Center, Rochester, NY USA; 142 Department of Pediatrics, University of Florida College of Medicine, Jacksonville, FL USA; 143 Department of Pediatrics, University of Buffalo Jacobs School of Medicine and Biomedical Sciences, Buffalo, NY USA; 144 Department of Pediatrics, Children’s Minnesota, Minneapolis, MN USA; 145 Department of Obstetrics and Gynecology, Northwell Health and the Zucker School of Medicine at Hofstra/Northwell, New Hyde Park, NY USA; 146 Department of Science Education, Northwell Health and the Zucker School of Medicine at Hofstra/Northwell, New Hyde Park, NY USA; 147 Institute of Health System Science, Northwell Health, Feinstein Institutes for Medical Research, Manhasset, NY USA; 148 Department of Pediatrics, Children’s Healthcare of Atlanta Emory University, Atlanta, GA USA; 149 Department of Biostatistics, Vanderbilt University Medical Center, Nashville, TN USA; 150 Department of Obstetrics and Gynecology, Vanderbilt University Medical Center, Nashville, TN USA; 151 Department of Women’s Health, Henry Ford Health, Detroit, MI USA; 152 Department of Biostatistics and Epidemiology, Environmental and Occupational Health Sciences Institute, Rutgers University, Piscataway, NJ USA; 153 Center for Advanced Biotechnology & Medicine, Rutgers University, Piscataway, NJ USA; 154 Departments of Biochemistry and Microbiology & Anthropology, Rutgers University, New Brunswick, NJ USA; 155 Department of Pediatrics, Robert Wood Johnson Medical School, Rutgers University, New Brunswick, NJ USA; 156 Departments of Pediatrics, Family Medicine, and Community Health, Robert Wood Johnson Medical School, Rutgers University, New Brunswick, NJ USA; 157 Department of Obstetrics, Gynecology, and Reproductive Sciences, Robert Wood Johnson Medical School, Rutgers University, New Brunswick, NJ USA; 158 Department of Obstetrics and Gynecology, Saint Peter’s University Hospital, New Brunswick, NJ USA; 159 Department of Public Health Sciences, Medical University of South Carolina, Charleston, SC USA; 160 Department of Obstetrics and Gynecology, Medical University of South Carolina, Charleston, SC USA; 161 Department of Global and Community Health, George Mason University, Fairfax, VA USA; 162 Department of Pediatrics, Medical University of South Carolina, Charleston, SC USA; 163 Department of Biostatistics, Epidemiology and Informatics, Department of Obstetrics and Gynecology, University of Pennsylvania Perelman School of Medicine, Philadelphia, PA USA; 164 Division of Neonatology, Department of Pediatrics, Children’s Hospital of Philadelphia, University of Pennsylvania Perelman School of Medicine, Philadelphia, PA USA; 165 Division of Maternal-Fetal Medicine, Department of Obstetrics & Gynecology, Feinberg School of Medicine, Northwestern University, Chicago, IL USA; 166 Division of Neonatology, Department of Pediatrics, Ann & Robert H. Lurie Children’s Hospital, Feinberg School of Medicine, Northwestern University, Chicago, IL USA; 167 Division of Maternal-Fetal Medicine, Department of Obstetrics & Gynecology, Hackensack University Medical Center, Hackensack Meridian School of Medicine, Nutley, NJ USA; 168 Division of Neonatology, Department of Pediatrics, Hackensack University Medical Center, Hackensack Meridian School of Medicine, Nutley, NJ USA; 169 Division of Developmental and Behavioral Pediatrics, Department of Pediatrics, Hackensack University Medical Center, Hackensack Meridian School of Medicine, Nutley, NJ USA; 170 Department of Pathology, Feinberg School of Medicine, Northwestern University, Chicago, IL USA; 171 Division of Infectious Diseases, Department of Pediatrics, Ann & Robert H. Lurie Children’s Hospital, Feinberg School of Medicine, Northwestern University, Chicago, IL USA; 172 Division of Neonatology, Department of Pediatrics, Emory University School of Medicine and Cerebral Palsy Foundation, Atlanta, New York, NY USA; 173 Division of Epidemiology & Community Health, School of Public Health, University of Minnesota, Minneapolis, MN USA; 174 Division of Research & Evaluation, HealthPartners Institute, Minneapolis, MN USA; 175 Care Delivery Research, Allina Health, Minneapolis, MN USA; 176 Department of Obstetrics and Gynecology, University of Miami Miller School of Medicine, Miami, FL USA; 177 Department of Obstetrics, Gynecology and Reproductive Sciences, University of Miami Miller School of Medicine, Miami, FL USA; 178 Mailman Center for Child Development, University of Miami Miller School of Medicine, Miami, FL USA; 179 Department of Obstetrics, Gynecology and Reproductive Sciences and Department of Public Health Sciences, University of Miami School of Medicine, Miami, FL USA; 180 Department of Pediatrics, University of Miami Miller School of Medicine, Miami, FL USA; 181 School of Nursing and Health Studies, University of Miami, Miami, FL USA; 182 Psychiatry and Behavioral Sciences, MIND Institute, University of California Davis, Sacramento, CA USA; 183 Center for Perinatal Research, Abigail Wexner Research Institute and Division of Neonatology, Nationwide Children’s Hospital and Department of Pediatrics, College of Medicine and Division of Epidemiology, College of Public Health, The Ohio State University, Nationwide Children’s Hospital and The Ohio State University, Columbus, OH USA; 184 Center for Biobehavioral Health, Abigail Wexner Research Institute, Nationwide Children’s Hospital and Department of Pediatrics, College of Medicine and Division of Epidemiology, College of Public Health, The Ohio State University, Nationwide Children’s Hospital and The Ohio State University, Columbus, OH USA; 185 Division of Maternal-Fetal Medicine, Department of Obstetrics and Gynecology, College of Medicine and Division of Epidemiology, College of Public Health, The Ohio State University, Columbus, OH USA; 186 Center for Precision Environmental Health and Department of Medicine, Baylor College of Medicine, Houston, TX USA; 187 Department of Family and Community Medicine, University of Texas Health Science Center at Houston (UTHealth Houston) McGovern Medical School, Houston, TX USA; 188 Department of Obstetrics, Gynecology and Reproductive Sciences, University of Texas Health Science Center at Houston (UTHealth Houston) McGovern Medical School, Houston, TX USA; 189 Department of Pediatrics, University of Texas Health Science Center at Houston (UTHealth Houston) McGovern Medical School, Houston, TX USA; 190 Department of Epidemiology, Geisel School of Medicine at Dartmouth, Hanover, NH USA; 191 Departments of Psychiatry, Pediatrics & Epidemiology, Geisel School of Medicine at Dartmouth, Dartmouth Hitchcock Medical Center, Hanover, NH USA; 192 Community Environmental Health Program, Department of Pharmaceutical Sciences, College of Pharmacy, University of New Mexico Health Sciences Center, Albuquerque, NM USA; 193 Center for Development and Disability, University of New Mexico, Albuquerque, NM USA; 194 Community Environmental Health Program, Department of Pharmaceutical Sciences UNM, College of Pharmacy, University of New Mexico Health Sciences Center, University of Chicago, Albuquerque, Chicago, IL USA; 195 Department of Psychiatry and Behavioral Sciences, University of California, San Francisco, San Francisco, CA USA; 196 Department of Internal Medicine, Comprehensive Cancer Center, University of New Mexico Health Sciences Center, Albuquerque, NM USA; 197 Department of Pediatrics, Department of Psychiatry and Human Behavior, Warren Alpert Medical School of Brown University, Providence, RI USA; 198 Department of Environmental Health, Rollins School of Public Health, Emory University, Atlanta, GA USA; 199 Department of Psychiatry and Human Behavior, Warren Alpert Medical School of Brown University, Providence, RI USA; 200 Department of Pediatrics, Warren Alpert Medical School of Brown University, Providence, RI USA; 201 Department of Pediatrics, Thompson Center for Autism & Neurodevelopment, University of Missouri, Columbia, MO USA; 202 Department of Pediatrics, Children’s Mercy-Kansas City, Kansas City, MO USA; 203 Department of Pediatrics, Wake Forest School of Medicine, Winston-Salem, NC USA; 204 Department of Pediatrics, University of Hawaii John A Burns School of Medicine, Honolulu, HI USA; 205 Department of Pediatrics, UCLA Clinical and Translational Science Institute at The Lundquist Institute, Harbor-UCLA Medical Center, Los Angeles, CA USA

**Keywords:** Vaginal microbiota structure, Pregnancy, Host factors, 16S rRNA gene amplicon sequence data, Meta-analysis, ECHO cohort, Microbial communities, Policy and public health in microbiology, Biomarkers, Medical research, Risk factors

## Abstract

Using pooled vaginal microbiota data from pregnancy cohorts (N = 683 participants) in the Environmental influences on Child Health Outcomes (ECHO) Program, we analyzed 16S rRNA gene amplicon sequences to identify clinical and demographic host factors that associate with vaginal microbiota structure in pregnancy both *within* and *across* diverse cohorts. Using PERMANOVA models, we assessed factors associated with vaginal community structure in pregnancy, examined whether host factors were conserved across populations, and tested the independent and combined effects of host factors on vaginal community state types (CSTs) using multinomial logistic regression models. Demographic and social factors explained a larger amount of variation in the vaginal microbiome in pregnancy than clinical factors. After adjustment, lower education, rather than self-identified race, remained a robust predictor of *L. iners* dominant (CST III) and diverse (CST IV) (OR = 8.44, 95% CI = 4.06–17.6 and OR = 4.18, 95% CI = 1.88–9.26, respectively). In random forest models, we identified specific taxonomic features of host factors, particularly urogenital pathogens associated with pregnancy complications (*Aerococcus christensenii* and *Gardnerella* spp.) among other facultative anaerobes and key markers of community instability (*L. iners*). Sociodemographic factors were robustly associated with vaginal microbiota structure in pregnancy and should be considered as sources of variation in human microbiome studies.

## Introduction

Our understanding of the relationship of the human microbiome to health has expanded greatly, including its role in a range of conditions with developmental origins^1–6^. The vaginal microbiome, in particular, has been associated with clinical outcomes such as infections in the reproductive tract and preterm birth, although not all studies have been consistent^7–10^. Beyond the long-term health sequelae associated with preterm birth, the human microbiome in pregnancy shapes the acquisition of the infant microbiome, which is seeded at birth and undergoes rapid assembly in early life with implications across the life course^1–4^. The vertical transmission of vaginal microbiota may play a role in the acquisition of both the offspring gut microbiome^11^ and immune development with effects on airway allergic responses^2,4,12^.

Both culture and culture-independent methods have demonstrated that *Lactobacillus* spp. dominate the vaginal microbiome in many (but not all) women^13,14^. *Lactobacillus* spp. dominance has been consistently associated with the lowest levels of genital tract inflammation across various populations studied^13,15,16^. Among some women, vaginal microbiota appear to be highly dynamic communities that are relatively stable over time^17^, particularly during pregnancy, with increased proliferation of *L. crispatus* and decreased diversity^18–21^.

Host factors may shape the microbiome in pregnancy and could provide insight into modifiable targets for intervention. However, few studies have evaluated environmental and clinical factors shaping the structure of the human vaginal microbiome across multiple diverse cohorts. Furthermore, integrating the microbiome into population-based research across multiple studies is challenging because of the variety of methods used to assess microbial communities. Results of microbiome studies in relation to preterm birth, for example, have been variable across populations, which may be driven by differences in host characteristics.

Although host factors have been shown to confound gut microbiome studies^22^, relatively little is known about host factors and the vaginal microbiome in pregnancy. One exception is self-reported race^14,23^, which has been related to community state types (CSTs) in single-cohort studies^14^. Specifically, individuals who self-identify as Black are more likely to exhibit diverse microbial profiles than those who identify as White^24,25^. Although consistently documented, the social patterning of microbiome composition research to date has not accounted for potential host confounders or the ways in which it self-identified race may be a proxy for unmeasured host exposures. Studies of large and diverse populations are thus needed to disentangle the many social determinants of health that are closely linked to self-reported race.

Meta-analysis can be used to overcome sources of bias in single site studies. However, aggregating microbiome studies can be difficult due to the heterogeneity in methods for sample collection, DNA isolation, and DNA sequencing, as well as varying bioinformatics approaches. With the recent advent of publicly available large 16S gene amplicon libraries, understanding how host factors may affect the vaginal microbiome in pregnancy will facilitate the precision and validity of large-scale population-based studies^22,26^. Therefore, we sought to identify host factors associated with vaginal microbiome structure in pregnancy by leveraging existing 16S rRNA gene amplicon sequence data from a diverse set of cohorts with well-characterized clinical, demographic, and biological data.

## Results

### Participant and sample characteristics

We utilized vaginal 16S rRNA gene sequence data from samples collected from the National Institutes of Health-funded Environmental influences on Child Health Outcomes (ECHO) cohort**,** which includes caregivers and children participating in multiple existing longitudinal birth cohort studies. ECHO was designed to evaluate the impact of early life exposures on child health outcomes and includes survey, medical record, and biospecimen collections^27,28^, as well as a subset of cohorts with vaginal 16S rRNA gene sequence data (Table 1).

**Table 1:** Overview of cohorts and collected samples.

Cohort	Sample type and collection	Sequencing, DNA isolation
MARCH (U-M sites) Recruited from University of Michigan clinics	Self-collected vaginal Starplex star dual-headed swabs at 3 times across gestation beginning at 7 to > 28 weeks gestation Clinic sample preserved in All-Protect upon collection	16S rRNA gene (V4 region) Qiagen PowerMicrobiome DNA/RNA EP kit
MARCH (non-U-M sites) Recruited from 9 other non-UM sites across Michigan	Self-collected vaginal Starplex star dual-headed swabs and fecal (no preservative) > 28 weeks gestation Mail collection	16S rRNA gene (V4 region) Qiagen/Mobio power soil DNA extraction kit
Atlanta Recruited from prenatal public/private clinics in Atlanta	Vaginal (self-collected, mid-vaginal), along with oral and rectal Epicentre Catchall swabs, collected at 8–14 and 24–30 weeks gestation	16S rRNA gene (V3–V4 region) Qiagen/Mobio power soil DNA extraction kit
CREW (WISC and MAAP) WISC recruited farm and rural-nonfarm families in Wisconsin. MAAP recruited families in Detroit	Vaginal-rectal swabs sampled from clinic at > 28 weeks gestation	16S rRNAgene (V4 region) SequalPrep Normalization Plate Kit (Thermo Fisher Scientific)

*ATLANTA*  Emory Atlanta African American Maternal-Child Cohort, *MAAP* Microbes, Allergy, Asthma, and Pets, *MARCH* Michigan Archive for Research on Child Health, *WISC* Wisconsin Infant Study Cohort.

Overall, we analyzed vaginal 16S sequence data from 683 pregnant participants across several geographically distinct areas of the United States. Based on self-report, 63% of the participants were non-White (of whom 97% self-identified as Black); 37% were White; and 1.9% were Hispanic. The distribution differed across the ECHO participating cohorts along with receipt of public insurance and maternal education (Table 2). The mean age was 28 years (SD = 5.4), 43% had a normal body mass index (≥ 18.5 and < 25 kg/m^2^); 42% were nulliparous; and 12% smoked during pregnancy. Antibiotic use in pregnancy was common (38%) in all trimesters of pregnancy. Approximately 12% of women had hypertension during pregnancy; 3.7% were diagnosed with gestational diabetes; and 11% had a preterm birth (Table 2).

**Table 2:** Characteristics by ECHO cohort.

	Overall, N = 683	Atlanta, N = 396	MAAP, N = 48	MARCH, N = 123	WISC, N = 116	p-value
Community state type						< 0.001
* Non-L. iners Lactobacillus* dominant (I, II, V)	180 (26%)	60 (15%)	14 (29%)	59 (48%)	47 (41%)	
* L. iners* dominant (III)	294 (43%)	188 (48%)	15 (31%)	42 (34%)	49 (42%)	
Diverse (IV-B, IV-C)	207 (30%)	146 (37%)	19 (40%)	22 (18%)	20 (17%)	
(Missing)	< 5	< 5	0	0	0	
Self-reported race*						< 0.001
Non-White	428 (63%)	396 (100%)	15 (32%)	16 (13%)	1 (0.9%)	
White	250 (37%)	0 (0%)	32 (68%)	106 (87%)	112 (99%)	
Unknown or (missing)	5	0	< 5	< 5	< 5	
Hispanic						0.002
Hispanic	13 (1.9%)	3 (0.8%)	3 (7.7%)	6 (4.9%)	1 (0.9%)	
Non-Hispanic	661 (98%)	393 (99%)	36 (92%)	117 (95%)	115 (99%)	
(Missing)	9	0	9	0	0	
Maternal age						< 0.001
Mean (SD)	27.7 (5.4)	25.4 (4.8)	30.4 (3.9)	31.4 (4.8)	30.8 (3.7)	
(Missing)	< 5	< 5	0	< 5	0	
Education						< 0.001
BA or higher	253 (37%)	65 (16%)	21 (44%)	85 (70%)	82 (71%)	
HS/GED	171 (25%)	153 (39%)	< 5 (8.3%)	8 (6.6%)	6 (5.2%)	
Less than HS	64 (9.4%)	59 (15%)	< 5 (4.2%)	< 5 (2.5%)	0 (0%)	
Some college	193 (28%)	119 (30%)	21 (44%)	26 (21%)	27 (23%)	
(Missing)	< 5	0	0	1	1	
Public insurance						< 0.001
No	229 (41%)	88 (22%)	42 (93%)	99 (81%)	NA	
Yes	334 (59%)	308 (78%)	< 5 (6.7%)	23 (19%)	NA	
(Missing)	120	0	< 5	1	116	
Private insurance						< 0.001
No	320 (57%)	308 (78%)	6 (13%)	6 (4.9%)	NA	
Yes	243 (43%)	88 (22%)	39 (87%)	116 (95%)	NA	
(Missing)	120	0	< 5	1	116	
Gestational diabetes						0.6
No	604 (96%)	383 (97%)	NA	115 (95%)	106 (95%)	
Yes	24 (3.8%)	13 (3.3%)	NA	6 (5.0%)	5 (4.5%)	
(Missing)	55	0	48	< 5	5	
Antibiotics ever in pregnancy						< 0.001
No	322 (62%)	215 (54%)	NA	107 (87%)	NA	
Yes	196 (38%)	180 (46%)	NA	16 (13%)	NA	
(Missing)	165	< 5	48	0	116	
Antibiotics in first trimester						< 0.001
No	428 (83%)	310 (78%)	NA	118 (96%)	NA	
Yes	90 (17%)	85 (22%)	NA	5 (4.1%)	NA	
(Missing)	165	< 5	48	0	116	
Antibiotics in second trimester						0.001
No	436 (84%)	321 (81%)	NA	115 (93%)	NA	
Yes	82 (16%)	74 (19%)	NA	8 (6.5%)	NA	
(Missing)	165	< 5	48	0	116	
Antibiotics in third trimester						0.001
No	456 (88%)	338 (86%)	NA	118 (96%)	NA	
Yes	62 (12%)	57 (14%)	NA	5 (4.1%)	NA	
(Missing)	165	< 5	48	0	116	
Infant sex						0.7
Female	347 (51%)	204 (52%)	27 (56%)	57 (47%)	59 (51%)	
Male	335 (49%)	192 (48%)	21 (44%)	65 (53%)	57 (49%)	
(Missing)	< 5	0	0	< 5	0	
Smoking in pregnancy						< 0.001
No	588 (88%)	322 (82%)	41 (85%)	116 (97%)	109 (98%)	
Yes	84 (12%)	71 (18%)	7 (15%)	4 (3.3%)	2 (1.8%)	
(Missing)	11	< 5	0	< 5	5	
Hypertension						< 0.001
No	551 (88%)	333 (84%)	0 (NA%)	110 (91%)	108 (97%)	
Yes	77 (12%)	63 (16%)	0 (NA%)	11 (9.1%)	3 (2.7%)	
(Missing)	55	0	48	< 5	5	
Early pregnancy BMI (kg/m^2^) category						0.075
Normal (≥ 18.5 & < 25)	272 (43%)	161 (41%)	10 (37%)	63 (52%)	38 (45%)	
Obese (≥ 30)	192 (31%)	138 (35%)	6 (22%)	25 (20%)	23 (27%)	
Overweight (≥ 25 & < 30)	143 (23%)	82 (21%)	11 (41%)	29 (24%)	21 (25%)	
Underweight (< 18.5)	22 (3.5%)	15 (3.8%)	0 (0%)	5 (4.1%)	2 (2.4%)	
(Missing)	54	0	21	< 5	32	
Parity category						< 0.001
< 1	291 (43%)	187 (47%)	19 (40%)	52 (43%)	33 (28%)	
1	221 (32%)	114 (29%)	23 (48%)	45 (37%)	39 (34%)	
2	101 (15%)	58 (15%)	< 5 (2.1%)	16 (13%)	26 (22%)	
> 3	69 (10%)	37 (9.3%)	5 (10%)	9 (7.4%)	18 (16%)	
(Missing)	< 5	0	0	< 5	0	

Data shown (except maternal age) are n (%).

*BA or higher* college degree or higher, *HS/GED* high school or general equivalency degree, *BMI* body mass index.

*Self-identified as a proxy for lived experiences.

Fisher’s exact test; Kruskal–Wallis rank sum test; Pearson’s chi-squared test.

Maternal factors varied by cohort (Table 2), most notably for race, as the Emory Atlanta African American Maternal-Child Cohort (hereafter the ‘Atlanta cohort’) is composed entirely of Black women, while fewer than 1% of the Wisconsin Infant Study Cohort (WISC) participants are Black. The subset of the Michigan Archive for Research with Mothers on Child Health (MARCH) cohort with vaginal microbiome data was 13% Black and 87% White. Samples were independently collected at each site using study-specific protocols, stored, isolated, and sequenced using a range of methods prior to bioinformatic processing. Table 1 lists details of the cohorts’ sample types and collection, DNA isolation method, and sequencing used.

### 16S rRNA gene amplicon data

**Figure 1:**
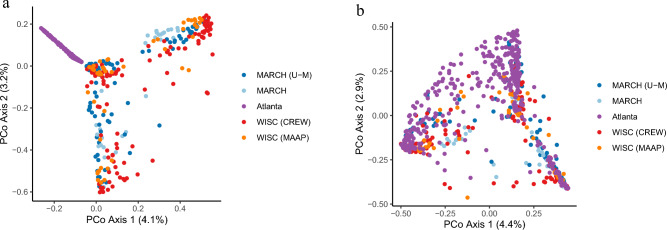
(**a**) Principal coordinates (PCoA) analysis of Bray–Curtis distances between samples based on amplicon sequence variants (ASVs) and (**b**) phylotypes demonstrating that using phylogenetic placement of ASVs on a reference tree removed a large degree of variation by site.

After removing one low-quality sample, the total number of reads per sample ranged from 2629 to 406,377, with a mean of 63,704 (SD = 63,970). Reads from 683 samples were denoised into amplicon sequence variants (ASVs), from which a total of 5232 phylotypes were constructed after mapping to a common phylogenetic tree constructed from full-length vaginal 16S rRNA encoding alleles. As evident in ordination plots shown in Fig. 1A, B, phylogenetic placement of the sequences (Fig. 1B) removed much of the variation that was evident across sites prior to processing in MALiAmPi (Fig. 1A), although some of the remaining site variation may also be due to inherent differences in host factors across the cohorts.

### The prevalence of CSTs varied by cohort

**Figure 2:**
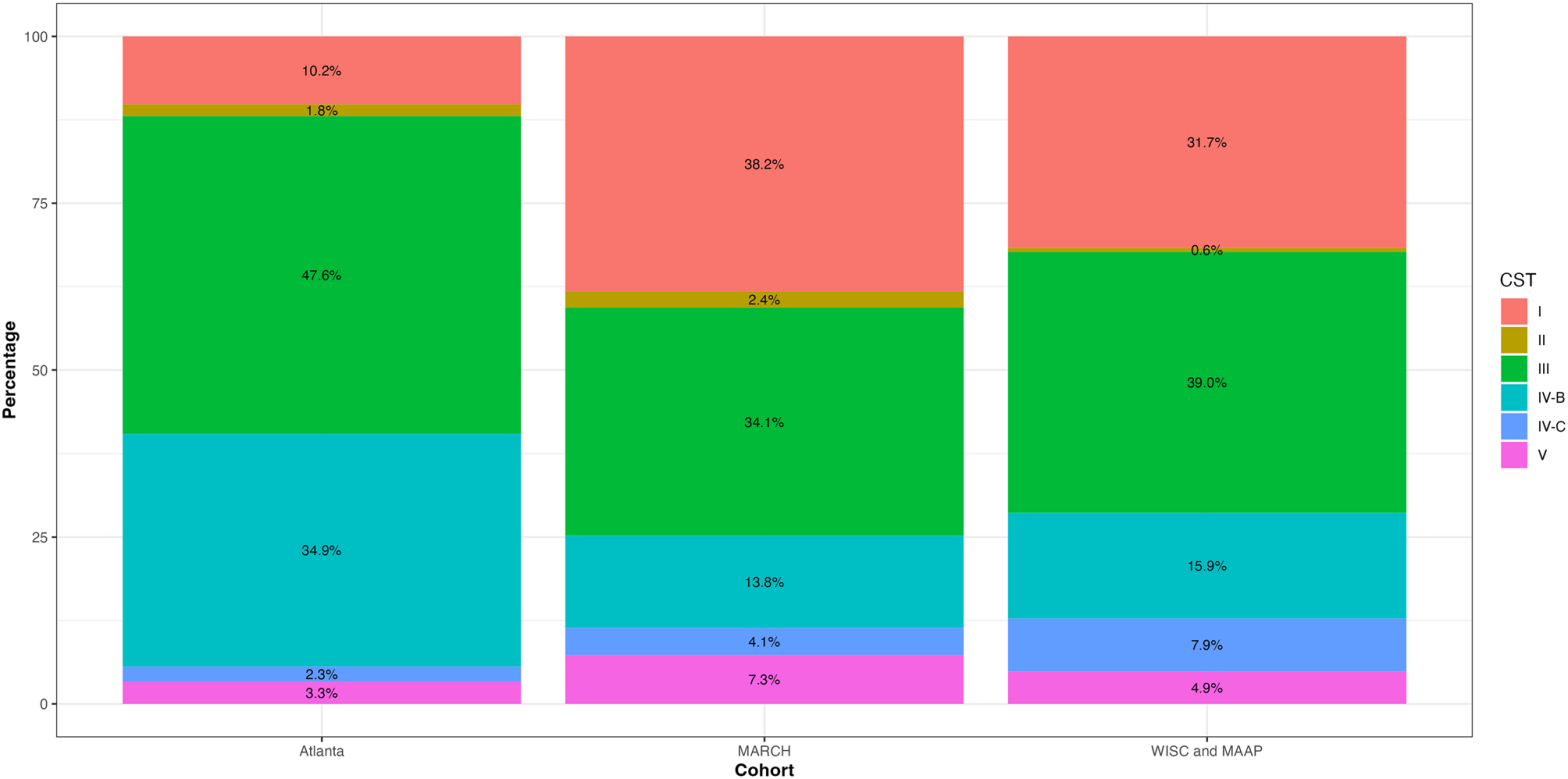
Community state types (CSTs) by ECHO cohort. CST I, *Lactobacillus crispatus* dominant; CST II, *L. gasseri* dominant; CST III, *L. iners* dominant; CST IV-B, diverse, characterized by high relative abundance of *Gardnerella* spp.*,* low relative abundance of *Candidatus Lachnocurva vaginae* (formerly known as *BVAB1*), and moderate relative abundance of *Fannyhessea vaginae *(previously *Atopobium vaginae*); CST IV-C, diverse array of facultative and strictly anaerobic bacteria, with low relative abundance of *Lactobacillus* spp*., G. vaginalis, Fannyhessea vaginae*, and *Ca. L. vaginae*); CST V, *L. jenseni* dominant.

Vaginal phylotypes were classified using the VAginaL community state typE Nearest CentroId clAssifier (VALENCIA)^11^. Four CSTs were *Lactobacillus* dominant: CST I, *L. crispatus*; CST II, *L. gasseri*; CST III, *L. iners*; and CST V, *L. jenseni*. The remainder were diverse polymicrobial communities: CST IV-B, characterized by high *Gardnerella* spp., low *Candidatus Lachnocurva vaginae* (formerly known as *BVAB1*), and moderate relative abundance of *Fannyhessea vaginae* (previously known as *Atopobium vaginae*), and CST IV-C, characterized by a diverse array of facultative and anaerobic bacteria and low relative abundances of *Lactobacillus* spp*., G. vaginalis, Fannyhessea vaginae*, and *Ca. L. vaginae*. While almost all CSTs were found in each cohort, the prevalence of each CST significantly varied by cohort (Fig. 2). For the Atlanta cohort, the most common was CST III (47.6%), followed by CST IV-B (34. 9%), whereas in the MARCH cohort, the most prevalent was CST I (38.2%), followed by CST III (34.1%). Although CST III and CST I were also the most common in WISC, the distribution was distinct from that in the MARCH cohort (Fig. 2). In contrast, non-*L. iners Lactobacillus-*dominant CSTs were less prevalent in the Atlanta cohort (Fig. 2).

### Host factors associated with vaginal microbiota structure across cohorts

**Figure 3:**
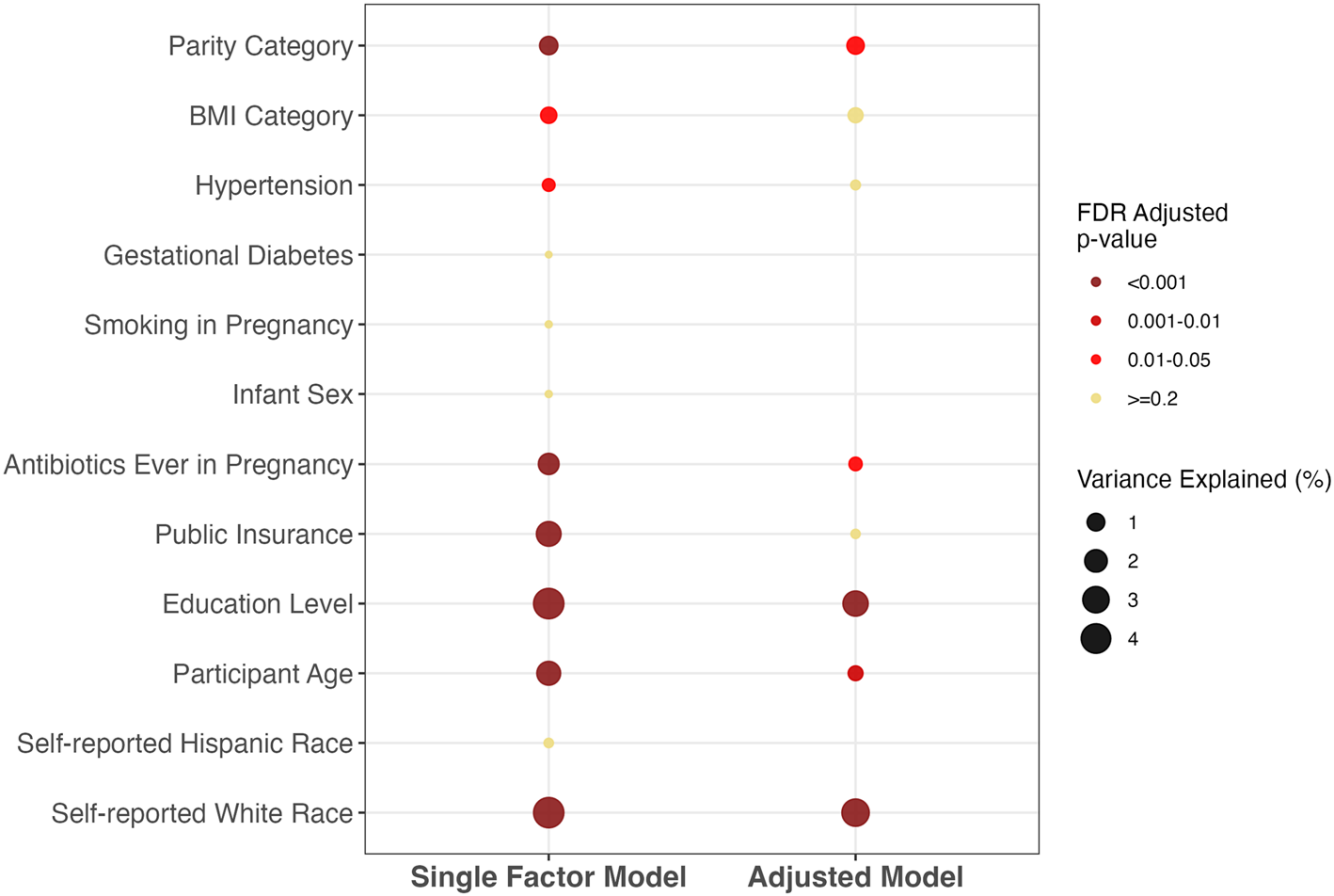
Single-adjusted (left) and multifactor-adjusted (left) permutational multivariate analysis of variance (PERMANOVA) pooled estimates of vaginal microbiota structure in pregnancy using Bray–Curtis distances across all cohorts. Only factors that were significantly associated with composition (p < 0.05) in single-factor models were retained for inclusion in the multifactor model using backward selection. *FDR* false discovery rate.

In single-factor, unadjusted permutational multivariate analysis of variance (PERMANOVA) models, both education and self-reported race accounted for the largest variance explained (4.28% and 4.26%, respectively; false discovery rate (FDR)-adjusted p-value < 0.001) in vaginal microbiota structure in pregnancy, followed by public insurance receipt (3% variance, FDR-adjusted p-value < 0.001) (Fig. 3). Antibiotics in pregnancy and age significantly contributed to 2% of the variance in vaginal microbiota structure (FDR-adjusted p-value < 0.01, each), while parity and smoking in pregnancy explained smaller amounts (Fig. 3). Although still significant after adjustment for multiple comparisons, early pregnancy BMI and hypertension each accounted for less than 1% of the variance in community structure. Self-reported Hispanic ethnicity, gestational diabetes, and sex of the infant were not associated with global community structure in pregnancy (Fig. 3).

### Host factors independently associated with vaginal microbiota structure

We next used multifactor PERMANOVA models to assess the independent effects of host factors on vaginal communities. In aggregate, host factors accounted for nearly 12% of the overall variance in vaginal microbiota structure in pregnancy. Education and self-reported race remained the most robust host factors associated with vaginal microbiota structure in pregnancy (4% of variance for each, FDR-adjusted p < 0.01) (Fig. 3). Parity, antibiotic use in pregnancy, and age remained independent predictors of vaginal microbiome structure in pregnancy but slightly less so after accounting for other host factors (FDR-adjusted p-values 0.01 to  < 0.05, respectively). The effects of early pregnancy BMI, hypertension, and public insurance receipt, in contrast, became attenuated after adjustment (Fig. 3).

### Host factors associated with vaginal microbiota structure were largely conserved across cohorts

**Figure 4:**
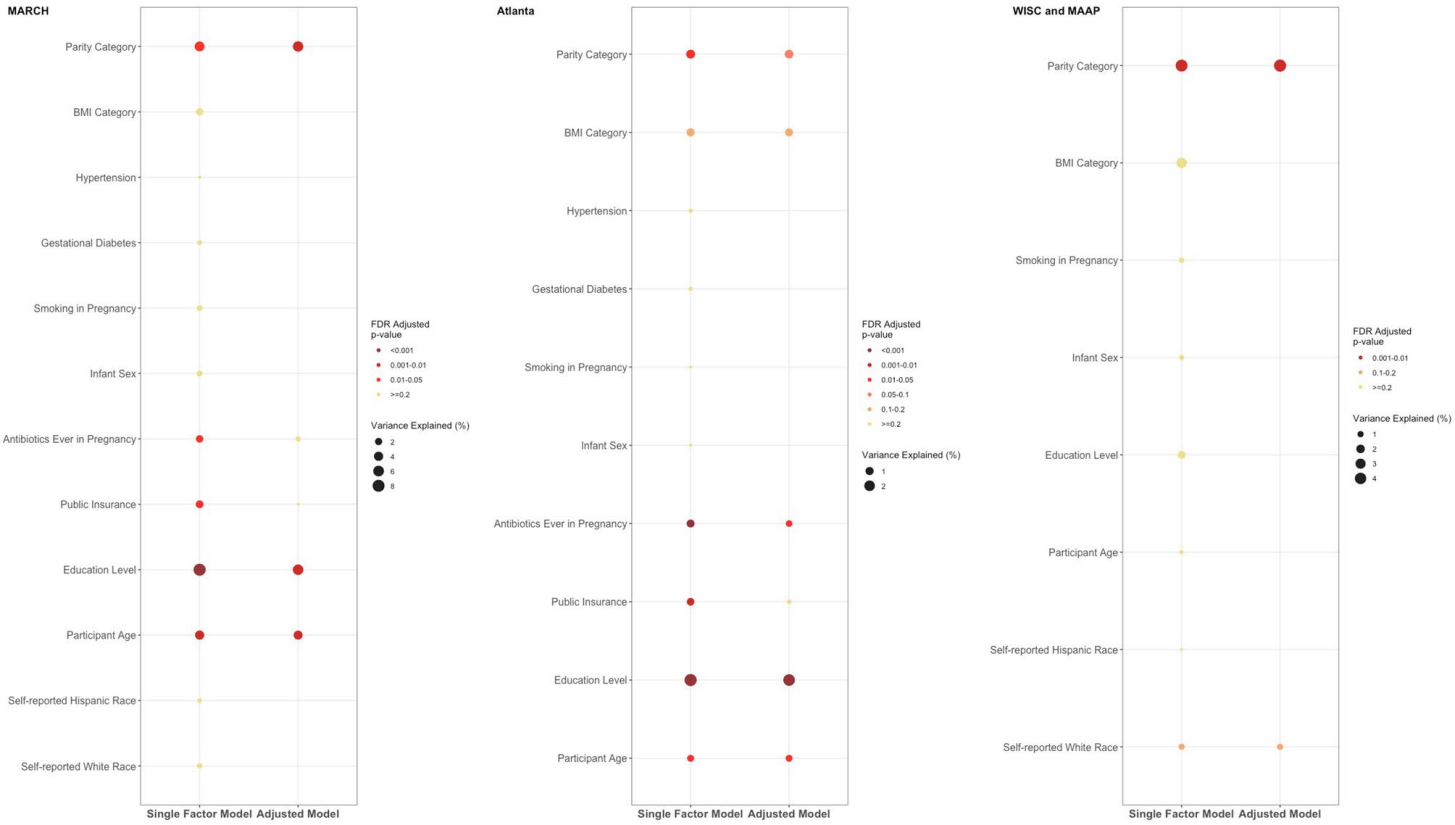
Associations between vaginal microbiota community structure and host factors across cohorts. Single-adjusted (left) and multifactor-adjusted (left) PERMANOVA estimates of vaginal microbiota structure in pregnancy using Bray–Curtis distances by individual cohort. Only factors that were significantly associated with composition (p < 0.05) in single-factor models were retained for inclusion in the multifactor model using backward selection.

We next generated cohort-specific PERMANOVA models to visualize results for each cohort independently (Fig. 4). The results were largely consistent, especially in the MARCH and Atlanta cohorts in which data on the host factors were well-aligned, further validating their independent effects on microbiome variation. Specifically, education and parity exhibited consistently robust associations with the vaginal microbiota community structure within each cohort (Fig. 4). Of note, self-reported race was significant in the pooled PERMANOVA model but not in the cohort-specific models, likely due to the substantial differences in its distribution between cohorts (Fig. 4 and Table 2). Some of the associations with host factors in the other cohorts were diminished in the WISC cohort, and others, such as antibiotic use in pregnancy, were not available.

### Taxonomic differences associated with robust host factors in pregnancy

**Figure 5:**
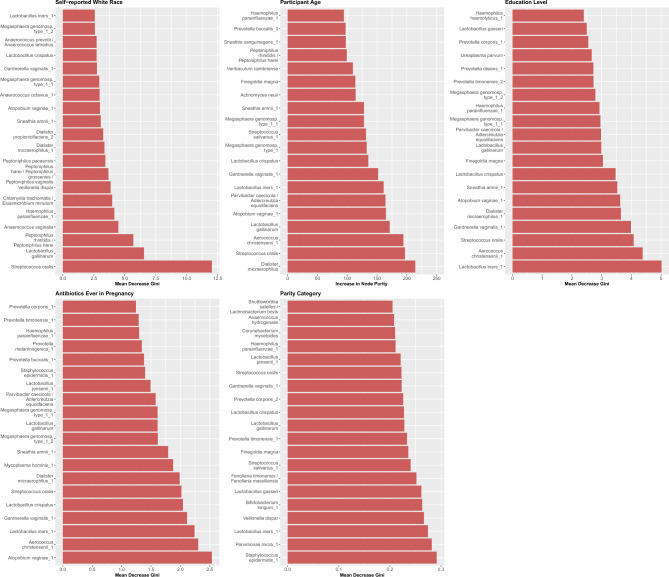
Random forest panel plots of the top ranked vaginal taxonomic features predictive of host factors.

We also examined taxonomic differences associated with host factors in pregnancy that may drive variation in vaginal microbiota structure. For each host factor that remained robust in the fully adjusted models of global community variance, we used random forest models to rank the most predictive phylotypes, which were classified to the species level. Of the taxa most predictive of educational attainment, *L. iners* was the most discriminant, followed by *A. christensenii*, *Streptococcus oralis*, and *G. vaginalis* (Fig. 5). The taxa most predictive of self-identified race were *S. oralis* followed by *Lactobacillus gallinarum* (Fig. 5). *Fannyhessea vaginae (previously Atopobium vaginae)* was the most predictive of antibiotic use in pregnancy followed by *A. christensenii*, *L. iners*, and *G. vaginalis* (Fig. 5*). Staphylococcus epidermidis*, *Parvimonas micra*, and *L. iners* were among the most predictive of parity. *Staphylococcus epidermidis*, *P. micra*, and *L. iners* were among the most predictive of parity, while *Dialister micraerophilus* was most predictive of host age (Fig. 5). Receiver operating curves (ROCs) for the top 20 taxa from the random forest models demonstrated areas under the curve (AUCs) ranging from 0.973 for self-identified race to 0.6114 for parity (Supplementary Fig. 1).

### Vaginal CSTs were associated with host factors

Due to their clinical relevance, we also tested the independent and joint associations between host factors and vaginal CSTs using a series of nested multinomial logistic regression models. CSTs were collapsed into three categories: *L. iners*-dominant (CST III), diverse (CST IV-B and IV-C) and non–*L. iners Lactobacillus-*dominant (i.e. CST I, II, V) CSTs, with the latter serving as the reference category. Prior to multivariable adjustment, we assessed the associations between host factors and CSTs for model selection (Supplementary Table 1). In the unadjusted model, self-identified White race was associated with a reduced odds of *L. iners* dominant (OR = 0.28, 95% CI = 0.19–0.42) and diverse CSTs (OR = 0.21, 95% CI = 0.14–0.32) compared to that of non-*L. iners Lactobacillus-*dominant CSTs (Table 3, Model 1). However, after adjustment for maternal education, the effect of race became attenuated and was no longer significant (Table3, Model 3). After adjustment for age, antibiotic use in pregnancy, and self-identified race, less than high school education was associated with *L. iners* dominant and diverse CSTs (OR = 7.81, 95% CI = 2.21–27.5 and OR = 8.84, 95% CI = 2.46–31.7, respectively) (Table 3). Other host factors that remained significantly associated with *L. iners* CSTs compared to non-*L. iners Lactobacillus-*dominant CSTs included parity and antibiotic use. Antibiotic use in pregnancy was also associated with increased odds of having both *L. iners*-dominant and diverse CSTs compared to non-*L. iners Lactobacillus-*dominant CSTs (CSTs (OR = 3.24 95% CI = 1.66–6.30 and OR = 4.24. 95% CI = 2.15–8.35, respectively).

**Table 3:** Host factors are independently associated with vaginal community state types in pregnancy.

	Model 1 (n = 680)						Model 2 (n = 680)						Model 3 (n = 680)					
	OR diverse CST	95% CI	p-value	OR L. iners CST	95% CI	p-value	OR diverse CST	95% CI	p-value	OR L. iners CST	95% CI	p-value	OR diverse CST	95% CI	p-value	OR L. iners CST	95% CI	p-value
White																		
No	–	–		–	–		–	–		–	–		–	–		–	–	
Yes	0.21	0.14, 0.32	< 0.001	0.28	0.19, 0.42	< 0.001	0.33	0.20, 0.55	< 0.001	0.43	0.28, 0.68	< 0.001	0.48	0.28, 0.82	0.007	0.61	0.38, 0.99	0.046
Maternal age							0.91	0.87, 0.96	< 0.001	0.92	0.88, 0.96	< 0.001	0.97	0.92, 1.02	0.2	0.97	0.93, 1.02	0.3
Education level																		
BA or higher													–	–		–	–	
HS/GED													3.48	1.73, 6.99	< 0.001	3.71	1.92, 7.17	< 0.001
Less than HS													8.46	2.60, 27.6	< 0.001	6.17	1.91, 19.9	0.002
Some college/assoc													2.58	1.43, 4.67	0.002	4.01	2.36, 6.82	< 0.001
Parity category																		
1 +																		
0																		
Antibiotics ever in pregnancy																		
No																		
Yes																		

	Model 4 (n = 680)						Model 5 (n = 516)											
	OR diverse CST	95% CI	p-value	OR L. iners CST	95% CI	p-value	OR diverse CST	95% CI	p-value	OR L. iners CST	95% CI	p-value						
White																		
No	–	–		–	–		–	–		–	–							
Yes	0.47	0.28, 0.81	0.006	0.59	0.37, 0.97	0.036	0.7	0.34, 1.43	0.3	0.7	0.36, 1.35	0.3						
Maternal age	0.96	0.91, 1.01	0.12	0.96	0.91, 1.01	0.093	0.97	0.90, 1.03	0.3	0.98	0.92, 1.05	0.6						
Education level																		
BA or higher	–	–		–	–		–	–		–	–							
HS/GED	3.4	1.69, 6.84	< 0.001	3.53	1.82, 6.86	< 0.001	5.2	2.22, 12.2	< 0.001	6.62	2.94, 14.9	< 0.001						
Less than HS	7.82	2.39, 25.6	< 0.001	5.43	1.67, 17.6	0.005	8.84	2.46, 31.7	< 0.001	7.81	2.21, 27.5	0.001						
Some college/assoc	2.52	1.39, 4.57	0.002	3.86	2.26, 6.58	< 0.001	4.18	1.88, 9.26	< 0.001	8.44	4.06, 17.6	< 0.001						
Parity category																		
1 +	–	–		–	–		–	–		–	–							
0	0.67	0.43, 1.06	0.09	0.55	0.36, 0.84	0.006	0.68	0.38, 1.19	0.2	0.55	0.32, 0.95	0.032						
Antibiotics ever in pregnancy																		
No							–	–		–	–							
Yes							4.24	2.15, 8.35	< 0.001	3.24	1.66, 6.30	< 0.001						

*OR* odds ratio, *CI* confidence interval, *BA or higher* college degree or higher, *HS/GED* high school or general equivalency degree.

Multinomial logistic regression models. Reference is non-*L. iners Lactobacillus* dominant (I, II, V) community state type.

Model 1 is adjusted for self-identified White race.

Model 2 is adjusted for self-identified White race, host age.

Model 3 is adjusted for self-identified White race, host age, educational attainment.

Model 4 is adjusted for self-identified White race, host age, educational attainment, parity.

Model 5 is adjusted for self-identified White race, host age, educational attainment, parity, and antibiotics use in pregnancy.

## Discussion

Our results identified new associations between microbiota structure and host factors overall as well as with specific taxonomic signatures. We also verified some associations, such as host self-identified race and age, that had been previously described and further tested their independent effects after adjustment for other factors. The most robust factors associated with vaginal microbiota structure in pregnancy were education, parity, and self-identified race, followed by prenatal antibiotic use. Together, education, age, self-identified race, antibiotic use, and parity explained a moderate amount of variance in prenatal vaginal community structure. Our results corroborate single-site studies linking vaginal community patterns and maternal education and age and parity^23,29–32^. They are also consistent with prior smaller studies on host socio-demographics and the structure of other microbial niches as well as those among non-pregnant populations.^33^

While self-identified race remained significantly associated with global microbiome structure, which was consistent with prior research,^14,25^, it was no longer significantly associated with vaginal community states in fully adjusted models. Race may reflect a range of unmeasured exposures, including maternal stress exposures related to discrimination and structural racism. In a recent paper, combined effects of individual and neighborhood-level measures of socioeconomic status were associated with vaginal microbiome composition^30^. Similar to our results, social host factors (i.e., education and self-identified race) were more closely related to the pregnancy microbiome than clinical factors (i.e., gestational diabetes, hypertension, early-pregnancy BMI, and antibiotics during pregnancy). These results suggest that exposures that occur over a longer period of time, compared to the relatively short period of gestation, have greater effects on the microbiota in pregnancy. Alternatively, social factors may be more influential because they reflect the physiological effects of multiple interacting host factors and unmeasured environmental factors, including urbanicity^34^, pollution, racial segregation, diet, and chronic stress^35,36^. Host factors associated with vaginal microbiota structure were also largely conserved across the MARCH and Atlanta cohorts, which are demographically and racially diverse.

Taken together, our results suggest that it is important to account for host factors in vaginal microbiota studies, as they may drive specific facets of community structure. The taxa most predictive of level of education, *L. iners*, is clinically relevant as a marker of instability of vaginal microbiome structure as well as bacterial vaginosis and pregnancy complications^37–40^. The other most discriminant taxa inversely associated with lower education were pathogens previously associated with pregnancy complications: *Aerococcus christensenii*, *S. oralis*, and *G. vaginalis*^41–44^*.* Taxa most discriminant of host factors across all cohorts were consistent with polymicrobial vaginal communities and bacterial vaginosis in contrast with the hallmarks of highly stable *L. crispatus*-dominant communities^15,45,46^, which confer pathogen resistance through the production of lactic acid and hydrogen peroxide by lowering vaginal pH and inflammation and which are critical for pregnancy maintenance. Our results also indicate that when phylogenetic distance is used to cluster and taxonomically classify 16S rRNA gene data, the effect of host factors on the vaginal community structure are remarkably consistent across populations. Furthermore, the distribution of CSTs varied by cohort, and such variation could be explained by the differential distribution of host factors, which should be considered in the design and analysis of future studies.

Strengths of the study include the large size and inclusion of well-characterized ECHO cohort metadata. We attempted to removed site-specific biases by using a common bioinformatic pipeline that included phylogenetic mapping and well-curated full-length 16S rRNA gene vaginal references. While we acknowledge that some technical sources of variation may have remained using this approach to harmonizing different cohorts’ 16S rRNA gene sequence data, the magnitude of variation across sites appeared to be less than it was prior to phylogenetic scaffolding. Limitations include some differences in sample collection protocols i.e., WISC and Microbes, Allergy, Asthma, and Pets (MAAP) samples, collected at group B streptococcus screenings in the third trimester, were drawn from recto-vaginal samples, which may have explained some of the differences between the host factors that remained consistent between the MARCH and Atlanta cohorts but were more attenuated in the WISC and MAAP cohort. There were also site differences in sequence length and primer kits, which may have resulted in some artifacts in the taxa we identified with host factors. In addition, limited data availability for some factors that likely affect vaginal microbiome structure precluded comparisons, such as prenatal antibiotic use in the WISC, as well as measures of diet, cohabitation/marital status, and douching practices^23^. Since data were sequenced prior to this study, we were not able to harmonize and account for the effect of gestational week at sample collection. Therefore, we aligned the sequence data to the first collection in pregnancy, although this varied from the first to the third trimester, which also may have explained some of the different results observed in the WISC and MAAP sites.

We disentangled host factors that may drive differences in microbial signatures across populations when their underlying distributions are differential. Our results suggest that host factors are plausible explanations for some of the inconsistent results in previous smaller studies, most of which, to date have failed to account for host factors. As such, our results have important implications for the design and analysis of future population-based studies of the vaginal microbiome in pregnancy and underscore the need to fully account for the complex relationships between host factors and the microbiome.

## Methods

### Study population

The ECHO cohort is a consortium of birth cohorts from across the United States designed to evaluate the impact of in utero and early-life exposures on child health outcomes and includes detailed survey, medical record, and bio-specimen collections. The design and purpose of the ECHO cohort have been previously described^27,28^. All ECHO cohorts with available vaginal samples that had previously undergone 16S rRNA gene amplicon sequencing were included in the present study (Table 1), which meta-analyzed 16S rRNA gene amplicon data where available (Supplementary Materials, Fig. 2) from ECHO across a diverse set of sites and populations. The Atlanta cohort participants were recruited between 8 and 14 weeks gestations from prenatal clinics affiliated with two hospitals in Atlanta, GA. The MAAP cohort recruited pregnant women during their second and third trimesters from two hospital systems in metro Detroit, MI, to understand how exposures in early life modify risk for asthma^47^. The Children’s Respiratory and Environmental Workgroup (CREW) includes both the WISC, drawn from rural medical centers in north-central Wisconsin^48^, and the MAAP cohort, drawn from urban metro-Detroit sites. The MARCH) a population-based cohort that recruited from initial prenatal appointments; for this study, we used a subset of the entire MARCH cohort that included both University of Michigan sites (MARCH U-M) and remote collection of samples from nine other sites across the state of Michigan. For this study, only those participants who provided informed consent for providing at least one vaginal swab sample during their pregnancy were included, and we analyzed the first sample collected in pregnancy.

### Clinical and demographic measures

At all sites, demographic data and health-related practices were collected from prenatal surveys, and health conditions, infection, delivery information, and antibiotic use were abstracted from the medical record. In MARCH, detailed information, including the infant’s sex, infant’s birth weight, complications of pregnancy, and pre-pregnancy BMI and gestational age, was derived from the birth certificate. Host factor metadata were harmonized to include self-reported White versus non-White race, any antibiotics in pregnancy, and public insurance as a proxy for socioeconomic status since household income was collected differently across sites.

### Vaginal sample collection

At the MARCH U-M sites, vaginal dual-headed dry swabs (Starplex™ Scientific S09D, Fisher Scientific) were self-collected in clinics. Immediately upon collection, AllProtect (Qiagen) preservative was added to the swabs, and they were stored for 24–48 h at −20 °C before being transported on ice for long-term storage at -80 °C. In the other MARCH sites, vaginal dual-headed dry swabs were self-collected at home, mailed to the laboratory, and archived at −80 °C. Similarly, in the Atlanta cohort, vaginal swabs were self-collected using the Sterile Catch-All Sample Swab (Epicentre), placed immediately in MoBio bead tubes, and transported on ice for archival storage at −80 °C. For both the MARCH and Atlanta cohorts, the first vaginal swab collected in pregnancy was analyzed. At the WISC and MAAP sites**,** vaginal/rectal swabs (Epicentre Catch-All) were collected by a provider within 6 weeks of delivery at the time of group B streptococcus screening. Swabs were stored in RNAlater (Thermo Fisher Scientific) at 4 °C for at least 24 h and then transferred to −80 °C. Prior to DNA extraction, swabs were thawed on ice and transferred to Lysing Matrix E (LME) tubes. RNAlater was transferred into a sterile tube and centrifuged at 16,000×*g* for 5 min at 4 °C. Pellets were re-suspended using cetyltrimethylammonium bromide buffer and transferred to the LME tube containing the swab^47^.

### DNA extraction, library preparation, and sequencing

#### MARCH

DNA was extracted from the MARCH U-M samples using the PowerMag kit (Qiagen; MoBio Laboratories) optimized for the epMotion 5075 TMX (Eppendorf). DNA samples were quantified using the Quant-iT PicoGreen dsDNA Assay kit (Thermo Fisher Scientific). The V4 region of the 16S rRNA gene was amplified using the dual-index sequencing strategy outlined in the MiSeq SOP^49^ at the MARCH U-M Microbiome Core^50^. Amplicons were sequenced using 250 bp Illumina MiSeq (MiSeq Reagent 222 kit V2) for 500 cycles according to the manufacturer’s instructions with modifications for the primer set. Libraries and sequencing reagents with custom read 1/read 2 and index primers added were prepared according to Illumina’s protocol for 2 nM libraries. The final load concentration was 4 pM, spiked with 15% PhiX.

DNA was extracted from the other MARCH samples also using the DNEasy Powersoil DNA Isolation kit (Qiagen) per the Human Microbiome Project’s protocol^51^ and MiSeq SOP^49^. Polymerase chain reaction (PCR) amplification was performed on the V4 region of the 16S rRNA gene following the mothur wet lab documentation^52^, using primer sets SB501-SB508 and SA701-SA712 ordered from IDT. Successfully amplified triplicate PCR reactions were pooled and purified using Agencourt AMPure XP (Beckman Coulter), and the concentration of 16S rRNA gene amplicons was quantified using the Quant-IT dsDNA assay kit (Invitrogen). The purified 16S rRNA gene pool was submitted to the Michigan State University Research Technology Support Facility Genomics Core for paired-end 250 bp sequencing on the Illumina MiSeq platform using V2 chemistry.

#### Atlanta

Samples underwent amplification of the V3-V4 region of the 16S rRNA gene following a two-step PCR protocol^53^. Amplicons were sequenced on the Illumina HiSeq 2500 modified to generate 300 bp paired-end reads. Additional details have been published elsewhere^8^.

#### WISC and MAAP

The V4 region of the 16S rRNA gene was amplified in triplicate reactions per sample using 515F/806R primers and PCR conditions previously described^12,54^. Pooled amplicon reactions with 5 ng were purified using the SequalPrep Normalization Plate Kit according to the manufacturer’s specifications, quantified using the Qubit dsDNA HS Assay Kit (Thermo Fisher Scientific), and pooled at equimolar concentrations. The amplicon library was constructed using the Agencourt AMPure XP system (Beckman-Coulter), quantified using the KAPA Library Quantification Kit (APA Biosystems), and diluted to 2 nM. Equimolar PhiX was added at 40% final volume to the amplicon library followed by sequencing on the Illumina NextSeq 500 platform employing a 2 × 150 bp sequencing run. Additional details have been published elsewhere for *WISC*^38^ and *MAAP*^32^.

### Bioinformatics

De-duplication was performed using the dual bar-code approach. We processed raw sequences from all sites together using the DADA2 Workflow for Big Data v.1.5.2 in order to cluster them into ASVs (https://benjjneb.github.io/dada2/bigdata.html)^55^ . For the Atlanta and MARCH cohorts, forward and reverse reads were trimmed using lengths of 255 and 225 bp, respectively, and filtered using a minimum quality score of 2. For the WISC and MAAP cohort, reads were maintained if they exhibited a maximum expected error of 2 and a read length of at least 150 bp. We then processed the ASVs using MaLiAmPi^56,57^, a computational tool designed to robustly combine 16S amplicon data for meta-analysis using phylogenetic placement. Sequences are mapped to a common tree, which we constructed from full-length 16S rRNA allele data from NCBI (cached as a repository on Zenodo^58^). We employed a minimum overlap at six for read-joining and removed Chimeras following the dada2 protocol.

Given that samples had previously undergone isolation and sequencing at each site (Table 1), we harmonized 16S rRNA gene sequences using MaLiAmPi^56,57^. We used the “refpkg.nf” module in MaLiAmPi to recruit alleles from this repository and assemble them de novo via RAxMLv8^59^. Finally, the amplicon sequence variants from DADA2^55^ were placed onto this phylogenetic tree via *pplacer*^60^, and metrics including alpha-diversity, pairwise phylogenetic distance, and taxonomic composition were derived via the *pplacer* utility *guppy*, per the *pplacer_place_classify.nf* module of MaLiAmPi.

After filtering of non-bacterial taxa, the relative abundance of a total of 5,232 phylotypes was estimated^61,62^. With mothur (version 1.48.0)^63,64^, we calculated Bray–Curtis distances and principal coordinates (PCoA) plotted with RStudio (version 2023.06.0 + 421), R (version 4.3.1), and the tidyverse^65^ (version 2.0.0) library, which includes ggplot2 (version 3.4.2). One sample with low counts was excluded. We also classified vaginal samples into CSTs using VALENCIA^11^, based on similarity to reference centroids.

### Statistical analysis

Demographic and clinical characteristics across cohorts are summarized in Table 2. To test for significant differences in the distribution of participant characteristics, we used chi-square or t-tests as appropriate. To identify host factors associated with vaginal microbiota structure in pregnancy, we generated single-factor PERMANOVA models based on Bray–Curtis distances overall and separately for each cohort using *adonis2* implementation in R’s vegan package and dispersion using *betsdispr* based on 100,000 permutations.

We also used multifactor PERMANOVA models to examine the independent effects of host factors using a backward variable selection approach. We adjusted p-values for multiple comparisons using a Benjamini and Hochberg FDR criterion of p < 0.05^66^. To test the independent effects of host factors on vaginal CSTs, we constructed a series of nested multinomial logistic regression models from the most robust predictors of vaginal microbiome variation identified from the PERMANOVA results. In these models, we collapsed the six VALENCIA classifications into three categories: non-*L. iners Lactobacillus* dominant (CSTs I, II, and V [reference]), *L. iners* dominant (CST III), and diverse (CST IV). Covariates were selected using a criterion of p < 0.2 in the PERMANOVA models including self-identified race, education level, maternal age, receipt of antibiotics in pregnancy, and parity category. Using a stepwise forward selection approach beginning with self-identified race, we compared parameter estimates as host factors were added to subsequent models to test whether the effect of self-identified race became attenuated after adjustment for each host factor or remained independently associated with vaginal CSTs. Models were run for the pooled cohort data as well as for each cohort individually using the R multinom() function from the nnet package.

We next examined how the factors retained in the multiple adjusted models associated with the relative abundance of specific taxa within communities. Using a machine learning approach utilizing a random forest classifier for each robust host factor in the PERMANOVA models, we ranked specific taxa that contributed the largest amount of homogeneity in the nodes and leaves of the forest trees by estimating the mean decrease in Gini index and increase in node purity coefficients for categorical and continuous predictors, respectively. Gini coefficients are estimated each time the tree is split on each feature, with higher values ranking greater discrimination. Random forest classifiers were run using a test and train validation set approach, splitting the data to set aside 20% for testing (80/20 split). We generated supervised random forest model plots and validated them using ROCs with AUCs using “randomForest” based on Breiman’s random forest algorithm for classification and regression^67^ and “pROC” package in R. Multidimensional scaling plots of the proximity matrix were also used to rank taxa between samples. Models were tuned focusing on the optimal number of variables randomly sampled as candidates at each split (“mtry”) and the optimal number of trees (“ntree”) in R. Default values for number of variables randomly sampled as candidates at each split were the square root of the number of variables in the model from 5,228 taxa. We set the hyperparameter nTree to 500, and tuned with “caret” until the out-of-bag error stopped decreasing.

Missing data in the outcome variables were imputed by calculating the variable median for the numeric variable and the mode for the categorical variables by cohort except when it was not missing at random (i.e., for an entire cohort). All statistical analyses were conducted using R version 4.2.3^68^. The University of Michigan IRB approved the research, which was deemed exempt and performed in accordance the Declaration of Helsinki. Informed consent was obtained from all participants.

### Supplementary Information

Supplementary Information 1.

Supplementary Information 2.

## Data Availability

The unprocessed 16S rRNA gene amplicon data are publicly available for the Atlanta cohort (https://www.ncbi.nlm.nih.gov/sra, PRJNA725416), MARCH (https://www.ncbi.nlm.nih.gov/sra, PRJNA1041860) and MAAP/WISC (European Nucleotide Archive: PRJEB46659). Select de-identified data from the ECHO Program are available through the Eunice Kennedy Shriver National Institute of Child Health and Human Development’s Data and Specimen Hub (DASH). Code for the study can be accessed publicly^69^. Information on study data not available on DASH can be obtained by contacting the ECHO Data Analysis Center at ECHO-DAC@rti.org with inquiries.
