# Supplementary material for: Host factors are associated with vaginal microbiome structure in pregnancy in the ECHO Cohort Consortium
Source: Sci Rep. 2024 May 23;14:11798. doi: 10.1038/s41598-024-62537-7 (PMC11116393; doi:10.1038/s41598-024-62537-7)
Supplement: Supplementary file 2 — Supplementary Information 2. [file 41598_2024_62537_MOESM2_ESM.docx]

**Supplementary Material**

**Host factors are associated with vaginal microbiome structure in pregnancy in the ECHO Cohort Consortium**

**Authors:** Kimberly McKee PhD, MPH^1*^, Christine M. Bassis PhD^2^, Jonathan Golob MD^2^, Beatrice Palazzolo MSc^1^, Ananda Sen PhD^1^, Sarah S. Comstock PhD^3^, Christian Rosas-Salazar MD, MPH^4^, Joseph B. Stanford MD^5^, Thomas O’Connor PhD^6^, James E. Gern MD^7^, Nigel Paneth MD, MPH^8^, Anne L. Dunlop MD, MPH^9^, for the Environmental influences on Child Health Outcomes (ECHO) Cohort Consortium

Supplementary Figure 1. Panel Plots of Receiver Operator Curves for Predicting Host Factors from Vaginal Taxonomic Features Trained and Tested in Random Forest Models


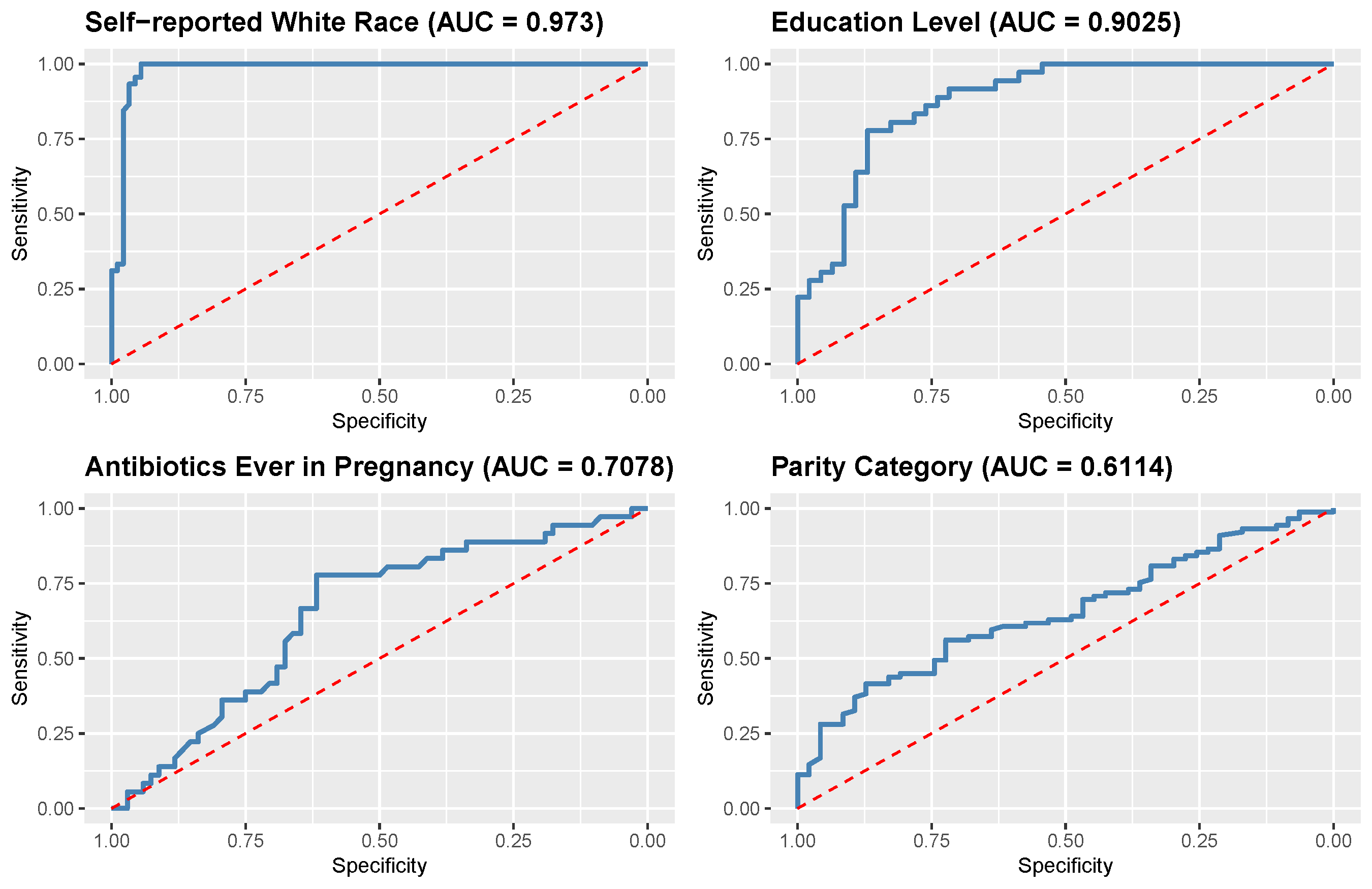


Supplementary Table 1: Associations between Host Factors and Community State Types

|  | | | | | |
| --- | --- | --- | --- | --- | --- |
|  | **Overall, N = 680** | **Diverse, N = 207** | **Lactobacillus iners (III), N = 293** | **Non-iners Lactobacillus (I, II, V), N = 180** | **p-value** |
| **Cohort** |  |  |  |  | <0.001 |
| Atlanta | 393 (58%) | 146 (71%) | 187 (64%) | 60 (33%) |  |
| MARCH | 123 (18%) | 22 (11%) | 42 (14%) | 59 (33%) |  |
| Wisconsin | 164 (24%) | 39 (19%) | 64 (22%) | 61 (34%) |  |
| **White** |  |  |  |  | <0.001 |
| 0 | 425 (63%) | 155 (75%) | 201 (69%) | 69 (39%) |  |
| 1 | 250 (37%) | 51 (25%) | 89 (31%) | 110 (61%) |  |
| (Missing) | 5 | 1 | 3 | 1 |  |
| **Hispanic** |  |  |  |  | 0.6 |
| Hispanic | 13 (1.9%) | 3 (1.5%) | 5 (1.7%) | 5 (2.8%) |  |
| Non-Hispanic | 658 (98%) | 201 (99%) | 286 (98%) | 171 (97%) |  |
| (Missing) | 9 | 3 | 2 | 4 |  |
| **Maternal Age** |  |  |  |  | <0.001 |
| Mean (SD) | 27.7 (5.4) | 26.5 (5.2) | 27.1 (5.2) | 30.3 (5.1) |  |
| (Missing) | 2 | 0 | 2 | 0 |  |
| **Education** |  |  |  |  | <0.001 |
| BA or Higher | 252 (37%) | 55 (27%) | 75 (26%) | 122 (68%) |  |
| HS/GED | 171 (25%) | 66 (32%) | 83 (29%) | 22 (12%) |  |
| Less than HS | 63 (9.3%) | 32 (15%) | 27 (9.3%) | 4 (2.2%) |  |
| Some College/Assoc. | 192 (28%) | 54 (26%) | 106 (36%) | 32 (18%) |  |
| (Missing) | 2 | 0 | 2 | 0 |  |
| **Public Insurance** |  |  |  |  | <0.001 |
| 0 | 228 (41%) | 55 (30%) | 82 (34%) | 91 (69%) |  |
| 1 | 332 (59%) | 131 (70%) | 161 (66%) | 40 (31%) |  |
| (Missing) | 120 | 21 | 50 | 49 |  |
| **Private Insurance** |  |  |  |  | <0.001 |
| 0 | 318 (57%) | 129 (69%) | 154 (63%) | 35 (27%) |  |
| 1 | 242 (43%) | 57 (31%) | 89 (37%) | 96 (73%) |  |
| (Missing) | 120 | 21 | 50 | 49 |  |
| **Antibiotics Ever in Pregnancy** |  |  |  |  | <0.001 |
| 0 | 320 (62%) | 83 (49%) | 134 (59%) | 103 (87%) |  |
| 1 | 195 (38%) | 85 (51%) | 95 (41%) | 15 (13%) |  |
| (Missing) | 165 | 39 | 64 | 62 |  |
| **Antibiotics in First Trimester** |  |  |  |  | <0.001 |
| 0 | 426 (83%) | 130 (77%) | 182 (79%) | 114 (97%) |  |
| 1 | 89 (17%) | 38 (23%) | 47 (21%) | 4 (3.4%) |  |
| (Missing) | 165 | 39 | 64 | 62 |  |
| **Antibiotics in Second Trimester** |  |  |  |  | <0.001 |
| 0 | 433 (84%) | 131 (78%) | 189 (83%) | 113 (96%) |  |
| 1 | 82 (16%) | 37 (22%) | 40 (17%) | 5 (4.2%) |  |
| (Missing) | 165 | 39 | 64 | 62 |  |
| **Antibiotics in Third Trimester** |  |  |  |  | 0.054 |
| 0 | 453 (88%) | 140 (83%) | 204 (89%) | 109 (92%) |  |
| 1 | 62 (12%) | 28 (17%) | 25 (11%) | 9 (7.6%) |  |
| (Missing) | 165 | 39 | 64 | 62 |  |
| **Birth Sex** |  |  |  |  | 0.6 |
| Female | 346 (51%) | 111 (54%) | 147 (50%) | 88 (49%) |  |
| Male | 333 (49%) | 96 (46%) | 145 (50%) | 92 (51%) |  |
| (Missing) | 1 | 0 | 1 | 0 |  |
| **Smoking in Pregnancy** |  |  |  |  | <0.001 |
| 0 | 587 (88%) | 170 (84%) | 245 (85%) | 172 (97%) |  |
| 1 | 82 (12%) | 33 (16%) | 43 (15%) | 6 (3.4%) |  |
| (Missing) | 11 | 4 | 5 | 2 |  |
| **Gestational Diabetes** |  |  |  |  | 0.4 |
| 0 | 602 (96%) | 178 (96%) | 267 (97%) | 157 (95%) |  |
| 1 | 23 (3.7%) | 8 (4.3%) | 7 (2.6%) | 8 (4.8%) |  |
| (Missing) | 55 | 21 | 19 | 15 |  |
| **Hypertension** |  |  |  |  | 0.014 |
| 0 | 548 (88%) | 161 (87%) | 232 (85%) | 155 (94%) |  |
| 1 | 77 (12%) | 25 (13%) | 42 (15%) | 10 (6.1%) |  |
| (Missing) | 55 | 21 | 19 | 15 |  |
| **BMI Category** |  |  |  |  | 0.019 |
| Normal (>=18.5 & <25) | 271 (43%) | 77 (40%) | 115 (41%) | 79 (50%) |  |
| Obese (>=30) | 191 (31%) | 68 (36%) | 90 (32%) | 33 (21%) |  |
| Overweight (>=25 & <30) | 142 (23%) | 40 (21%) | 59 (21%) | 43 (27%) |  |
| Underweight (<18.5) | 22 (3.5%) | 6 (3.1%) | 14 (5.0%) | 2 (1.3%) |  |
| (Missing) | 54 | 16 | 15 | 23 |  |
| **Parity Category** |  |  |  |  | 0.008 |
| 1 | 220 (32%) | 55 (27%) | 95 (33%) | 70 (39%) |  |
| 2 | 101 (15%) | 34 (16%) | 53 (18%) | 14 (7.8%) |  |
| 3+ | 69 (10%) | 26 (13%) | 30 (10%) | 13 (7.2%) |  |
| No prior | 289 (43%) | 92 (44%) | 114 (39%) | 83 (46%) |  |
| (Missing) | 1 | 0 | 1 | 0 |  |
| n (%) | | | | | |
| Pearson's Chi-squared test; Fisher's exact test; Kruskal-Wallis rank sum test | | | | | |

BA or higher = college degree or higher, HS/GED = high school or general equivalency degree, MARCH = Michigan Archive for Research on Child Health

Supplementary Figure 2. Flowchart of Sample Selection.

ECHO pregnancy with data availability on vaginal samples collected during pregnancy, sequenced and available for analysis

N=894 from 3 cohorts

**Excluded:** RNAseq sequence data/ 16S rRNA gene amplicon data not available (1 cohort)

1 cohort

N=680 from 3 cohorts (MARCH, Atlanta, MAAP+CREW-WISC)

**Excluded:**  quality filtering; any samples without host metadata
